# ITGBL1 promotes anoikis resistance and metastasis in human gastric cancer via the AKT/FBLN2 axis

**DOI:** 10.1111/jcmm.18113

**Published:** 2024-02-08

**Authors:** Kanger Shen, Wei Xia, Kun Wang, Juntao Li, Wei Xu, Haoran Liu, Kexi Yang, Jinghan Zhu, Jiayu Wang, Qinhua Xi, Tongguo Shi, Rui Li

**Affiliations:** ^1^ Jiangsu Institute of Clinical Immunology, The First Affiliated Hospital of Soochow University Suzhou China; ^2^ Jiangsu Key Laboratory of Clinical Immunology Soochow University Suzhou China; ^3^ Department of Gastroenterology The First Affiliated Hospital of Soochow University Suzhou China

**Keywords:** anoikis, FBLN2, gastric cancer, ITGBL1, metastasis

## Abstract

The resistance to anoikis plays a critical role in the metastatic progression of various types of malignancies, including gastric cancer (GC). Nevertheless, the precise mechanism behind anoikis resistance is not fully understood. Here, our primary focus was to examine the function and underlying molecular mechanism of Integrin beta‐like 1 (ITGBL1) in the modulation of anoikis resistance and metastasis in GC. The findings of our investigation have demonstrated that the overexpression of ITGBL1 significantly augmented the resistance of GC cells to anoikis and promoted their metastatic potential, while knockdown of ITGBL1 had a suppressive effect on both cellular processes in vitro and in vivo. Mechanistically, we proved that ITGBL1 has a role in enhancing the resistance of GC cells to anoikis and promoting metastasis through the AKT/Fibulin‐2 (FBLN2) axis. The inhibition of AKT/FBLN2 signalling was able to reverse the impact of ITGBL1 on the resistance of GC cells to anoikis and their metastatic capability. Moreover, the expression levels of ITGBL1 were found to be significantly elevated in the cancerous tissues of patients diagnosed with GC, and there was a strong correlation observed between high expression levels of ITGBL1 and worse prognosis among individuals diagnosed with GC. Significantly, it was revealed that within our cohort of GC patients, individuals exhibiting elevated ITGBL1 expression and diminished FBLN2 expression experienced the worst prognosis. In conclusion, the findings of our study indicate that ITGBL1 may serve as a possible modulator of resistance to anoikis and the metastatic process in GC.

## 1 INTRODUCTION

Gastric cancer (GC) is a prevalent malignancy on a global scale, characterized by significant morbidity and mortality rates.^1^ Despite substantial advances in therapeutic approaches, the 5‐year survival rate of patients who have been diagnosed with Stage III and IV GC, is rather low.^2^ A variety of complications caused by distant metastasis are closely related to the poor prognosis of GC patients.^3^ Therefore, it is valuable to identify new targets and understand the mechanism of metastasis to prevent or slow the metastasis of GC.

As a prevalent kind of apoptosis that occurs during the process of cellular metastasis, anoikis is defined as a process in which cells undergo programmed death after leaving the extracellular matrix, and it is a barrier to metastasis of tumour cells.^4^, ^5^ The ability to resist anoikis is of utmost importance for the viability and persistence of metastatic tumour cells throughout the bloodstream, lymphatic system, and remote organs.^6^ Tumour cells develop resistance to anoikis through numerous mechanisms, such as integrin dysregulation, abnormal activation of multiple antiapoptotic and prosurvival pathways, epithelial‐mesenchymal transition (EMT), and the activity of multiple metabolic pathways.^7^ For example, the protein 14‐3‐3σ has increased expression levels in hepatocellular carcinoma (HCC) tissues, actively facilitates the resistance to anoikis and the metastatic behaviour of HCC cells through the EGFR/ERK1/2 axis.^8^ Additionally, TCF7L2 transcriptionally activates PLAUR and enhances the anoikis resistance.^9^ Nevertheless, there is a lack of comprehensive understanding regarding the molecular mechanisms that contribute to the development of anoikis resistance in GC.

Integrin beta‐like 1 (ITGBL1), encoding a protein comprising 10 integrin epidermal EGF‐like repeat domains, was initially isolated from a cDNA library derived from osteoblasts in 1999.^10^ Prior research has indicated a correlation between ITGBL1 and the advancement and spread of several forms of cancer, including liver,^11^ ovarian,^12^ breast^13^ and colorectal cancers^14^ as well as GC.^15^ Rongkun Li noted that the upregulated expression of ITGBL1 exhibited a favourable correlation with both the tumour‐node‐metastasis (TNM) stage and the occurrence of distant metastasis in GC patients.^16^ Moreover, the overexpression of ITGBL1 was found to enhance the proliferation and invasion of GC cells through the activation of the Akt signalling pathway.^15^ Nevertheless, the precise mechanisms underlying the role of ITGBL1 in conferring resistance to anoikis in GC cells remain little elucidated.

In this research, we effectively established GC cells that are anoikis‐resistant (AR) and found that ITGBL1 overexpression promoted but the suppression of ITGBL1 expression resulted in the inhibition of anoikis resistance and metastasis in GC cells through the AKT/FBLN2 pathway. Our findings demonstrate the critical role of ITGBL1 in controlling anoikis resistance and metastasis in GC.

## 2 MATERIALS AND METHODS

### 2.1 Bioinformatics analysis

The data pertaining to GC were acquired from the TCGA database and the GEPIA database. The datasets were sorted according to the relative expression level of ITGBL1 in GC and combined with the clinicopathological information of GC patients to evaluate the differential expression of ITGBL1 in cancerous and normal gastric tissues, as well as its relationship with stage. To identify the downstream molecules regulated by ITGBL1, a total of 375 GC patients from the TCGA database were divided into two groups, the high and low expression groups, using the median values of the ITGBL1 expression level as thresholds, with a cut‐off value of 0.92166570. Among the total, there were 187 cases in the group with high expression of ITGBL1 and 188 cases in the group with low expression. Next, differential expression analysis and weighted gene coexpression network analysis (WGCNA) were performed. *p* < 0.05 and |log2 (fold change)|>1 were considered to indicate differential gene expression. The soft threshold of *R* ^2^ > 0.9 was chosen as the most suitable value for designing the scale‐free network. After merging similar modules, the module with the highest correlation coefficient was selected for further analysis. The overlapping genes between the differentially expressed genes and the module genes were considered potential genes related to ITGBL1. Then, these potential genes were further screened using two machine learning techniques, least absolute shrinkage and selection operator (LASSO) and support vector machine (SVM), and the genes overlapping between the results of the two techniques were considered important ITGBL1‐related genes filtered from the TCGA database.

### 2.2 Clinical samples and immunohistochemical (IHC) analysis

Two independent GC tissue microarrays (TMAs, #HStmA180Su19 and #HstmAde150CS401) were purchased from Shanghai Outdo Biotech Co., Ltd. (Shanghai, China) and contained tumour tissue from 94 cases of M0 GC and tumour tissue from 75 cases of M1 GC. The TMAs and paraffin‐embedded sections of mouse lung tissue were subjected to incubation with anti‐ITGBL1 rabbit polyclonal antibody (Sigma–Aldrich, MO, USA, #HPA005676), anti‐FBLN2 rabbit polyclonal antibody (Novus Biologicals, CO, USA, #NBP1‐33479), anti‐AKT rabbit polyclonal antibody (Beyotime, Shanghai, China, #AA326) or anti‐P‐AKT rabbit polyclonal antibody (CST, MA, USA, #4060) at a temperature of 4°C for the duration of one night and were then incubated with HRP‐conjugated goat anti‐rabbit IgG as a secondary antibody for 1 h at 37°C. Two seasoned pathologists evaluated the immunohistochemistry score using a blinded process and the criteria and procedure provided below.

### 2.3 Cell culture

The human GC cell lines AGS, HGC27, MKN28 and MKN45, as well as the normal gastric mucosal cell line GES‐1, were obtained from the American Type Culture Collection (ATCC, Manassas, VA, USA). These cell lines were cultured in a humidified incubator at a temperature of 37°C with a CO_2_ concentration of 5%. The HGC‐27 cells were cultured in DMEM (EallBio, Beijing, China, #03.100‐6C), whereas the other cells were cultured in RPMI‐1640 media (EallBio, #03.4007‐C). The culture medium mentioned above were enhanced with 10% fetal bovine serum (FBS, EallBio, #3. U16001DC) and 1% penicillin–streptomycin (NCM Biotech, Suzhou, China, #C100C‐5).

### 2.4 Western blot analysis

The cellular samples were gathered and subjected to lysis using RIPA lysis buffer (Beyotime, #P0013) supplemented with protease inhibitors and phosphatase inhibitors (Beyotime, #P1045). The protein concentration was determined using the BCA Protein Assay Kit (Beyotime, #P0011). The total amount of protein (30 μg) was subjected to separation using a 10% SDS–PAGE gel (NCM Biotech, #P2012) and afterwards transferred onto PVDF membranes with a pore size of 0.45 μm (GE Healthcare Life Science, Germany, #10600023). The membranes were obstructed using a 5% bovine serum albumin (BSA) solution (Fcmacs, Nanjing, China, #FMS‐WB021) for a duration of 1.5 h. Subsequently, the membranes were subjected to incubation with the specified primary antibodies at a temperature of 4°C for an overnight period. The next day, the membranes underwent a triple wash using TBST (1 × TBS, 0.1% Tween 20) followed by incubation with the appropriate HRP‐conjugated secondary antibodies for a duration of 1 h at room temperature. The membranes underwent three further washes using TBST. The visualization of immunoreactions was conducted using ECL reagents (NCM Biotech, #10,100) and a ChemiDocTM MP Imaging System (Bio‐Rad, CA, USA). The Table S1 provides all antibodies used for western blot analysis.

### 2.5 Forced suspension culture

Poly‐2‐hydroxyethyl methacrylate (poly‐HEMA) (Sigma–Aldrich, #P3932) was dissolved to 10 mg/mL in absolute ethanol in a 37°C water bath overnight. One millilitre of dissolved poly‐HEMA was added to a 6‐well plate and allowed to evaporate. After complete evaporation, the six‐well plates were sterilized by UV irradiation on an ultraclean table for 2 h before culture in suspension.

### 2.6 Construction of anoikis‐resistant cells

To obtain AR‐GC cells, GC cells were first cultured in suspension for 24 h, and the surviving cells were then cultured under adhesion conditions. The above steps were repeated four to five times, and the last surviving cells were considered anoikis‐resistant cells.

### 2.7 Apoptosis assays

The apoptosis assays were conducted using the PE Annexin V Apoptosis Detection Kit I (BD Biosciences, NJ, USA, #559763). To summarize, cellular samples were obtained and placed into flow tubes, subsequently, the samples were rinsed with PBS. Following this, the cells were resuspended in a 100 μL solution of 1× Binding Buffer, which was further supplemented with 5 μL of Annexin‐V‐PE and 7‐AAD. After a 20 min incubation period at room temperature without exposure to light, the rate of apoptosis was evaluated by flow cytometry (Beckman Coulter, CA, USA). Cells exhibiting positive staining for Annexin‐V and negative staining for 7‐AAD, as well as cells displaying positive staining for both Annexin‐V and 7‐AAD, were categorized as apoptotic cells.

### 2.8 Transwell migration and invasion assays

Transwell migration and invasion experiments were conducted using the approach described in our previous study.^17^ In the migration assay, a total of 3 × 10^4^ GC cells were seeded in the upper compartments of a 24‐well plate (Corning, NY, USA, #353097) with an 8 μm pore size membrane. The upper compartments were filled with 400 μL of serum‐free media, while the bottom compartments were filled with 400 μL of media containing 20% FBS. In the invasion assay, the Matrigel (Corning, #356234) was diluted with serum‐free media at a ratio of 1:15. Subsequently, the diluted Matrigel was added into the top compartments and allowed to solidify for a duration of 30 min. After 48 h of incubation, the migrated or invaded cells were fixed, stained, and photographed with an inverted microscope (Nikon, Tokyo, Japan).

### 2.9 Cell transfection and lentivirus infection

The commercial ITGBL1 siRNAs as well as the FBLN2 siRNAs were obtained from RiboBio (Guangzhou, China). The target sequences of the siRNAs are shown in Table S2. The plasmids containing FBLN2 cDNA and the respective control plasmids were procured from Miaolingbio (Wuhan, China). The cells were transfected with siRNAs or expression plasmids using Lipo8000™ Transfection Reagent (Beyotime, #C0533) following the manufacturer's specified instructions.

The lentiviruses expressing ITGBL1 cDNA, ITGBL1 short hairpin RNA (shRNA) with the sequence ITGBL1‐siRNA‐1, or FBLN2 shRNA with the sequence FBLN2‐siRNA‐2 were purchased from Shanghai GenePharma Co., Ltd. (Shanghai, China). The indicated cells were transduced with the corresponding lentiviral vectors (MOI: 40). After 72 h, cells were screened using puromycin dihydrochloride (Beyotime, #ST551).

### 2.10 RNA sequencing and bioinformatic analysis

Total RNA was extracted from ITGBL1‐overexpressing AR‐AGS cells and control cells using RNA isolater Total RNA Extraction Reagent (Vazyme, Nanjing, China, #R401‐01). RNA sequencing (RNA‐seq) analysis was performed by Shanghai Biotechnology Co., Ltd. (Shanghai, China).

The identification of differentially expressed genes (DEGs) was carried out based on the two criteria: (1)|log2 (fold change) | > 2 and (2) *p*‐Value <0.05. The heatmap and volcano plot were made using the R software, specifically utilizing the following packages: ‘pheatmap’, ‘ggplot2’ and ‘ggrepel’. The Kyoto Encyclopedia of Genes and Genomes (KEGG) pathway enrichment analyses were conducted using the R software, specifically utilizing the following packages: ‘clusterProfiler’, ‘org.Hs.eg.db’ and ‘pathview’. The DEGs are listed in Table S3.

### 2.11 Animal studies

The study utilized female NSG mice that were 6 weeks old and obtained from the Shanghai Laboratory Animal Center (Shanghai, China). A random assignment was used to allocate NSG mice into two groups: the sh‐NC group and the sh‐ITGBL1 group. Each group consisted of four mice. Mice were administered intravenous injections of sh‐NC or sh‐ITGBL1 AR‐MKN45 cells at a concentration of 2 × 10^6^ cells in 100 μL of PBS per mouse, through the tail vein. Following a duration of 8 weeks, the mice were euthanized, and the lung tissues were collected for further analysis. In the rescue trials, a random assignment was made of NSG mice to either the sh‐ITGBL1 group or the sh‐ITGBL1‐sh‐FBLN2 group, with a total of four mice in each group. The experimental procedure involved the administration of sh‐ITGBL1 or sh‐ITGBL1‐sh‐FBLN2 AR‐MKN45 cells to mice through intravenous injection into the tail vein. Each mouse received a dose of 2 × 10^6^ cells in 100 μL of PBS. After a duration of 6 weeks, the mice were euthanized, and the pulmonary tissues were collected for further analysis. After 2 weeks of fixation with 4% paraformaldehyde, haematoxylin–eosin staining was carried out. The enumeration of pulmonary metastatic nodules was conducted in a blinded fashion by two pathologists with extensive expertise in the field.

### 2.12 Haematoxylin–eosin staining and histological scoring

Histopathological investigation involved the utilization of a Haematoxylin and Eosin Staining Kit (Beyotime, #C0105) to perform tissue section staining. The lung tissues of mice were subjected to fixation using a 4% paraformaldehyde solution, followed by a series of washing, dehydration and embedding processes using paraffin. The specimens were sequentially sectioned into sections that were 5 μm in thickness. These sections were then subjected to staining with haematoxylin and eosin, which allowed for the visualization of nuclei and cytoplasm, respectively.

### 2.13 Statistical analysis

The statistical analysis of the data was conducted using GraphPad Prism (9.0). Student's *t*‐test was employed for data that exhibited a normal distribution, whereas the Wilcoxon rank‐sum test was utilized for data that did not conform to a normal distribution. The Pearson correlation analysis was employed to examine the association between the expression levels of ITGBL1 and FBLN2. The Kaplan–Meier method was employed for survival analysis. *p* > 0.05 was deemed to indicate statistical significance.

## 3 RESULTS

### 3.1 ITGBL1 is highly expressed in GC tissue and cells, and high ITGBL1 expression predicts a poor prognosis

**FIGURE 1:**
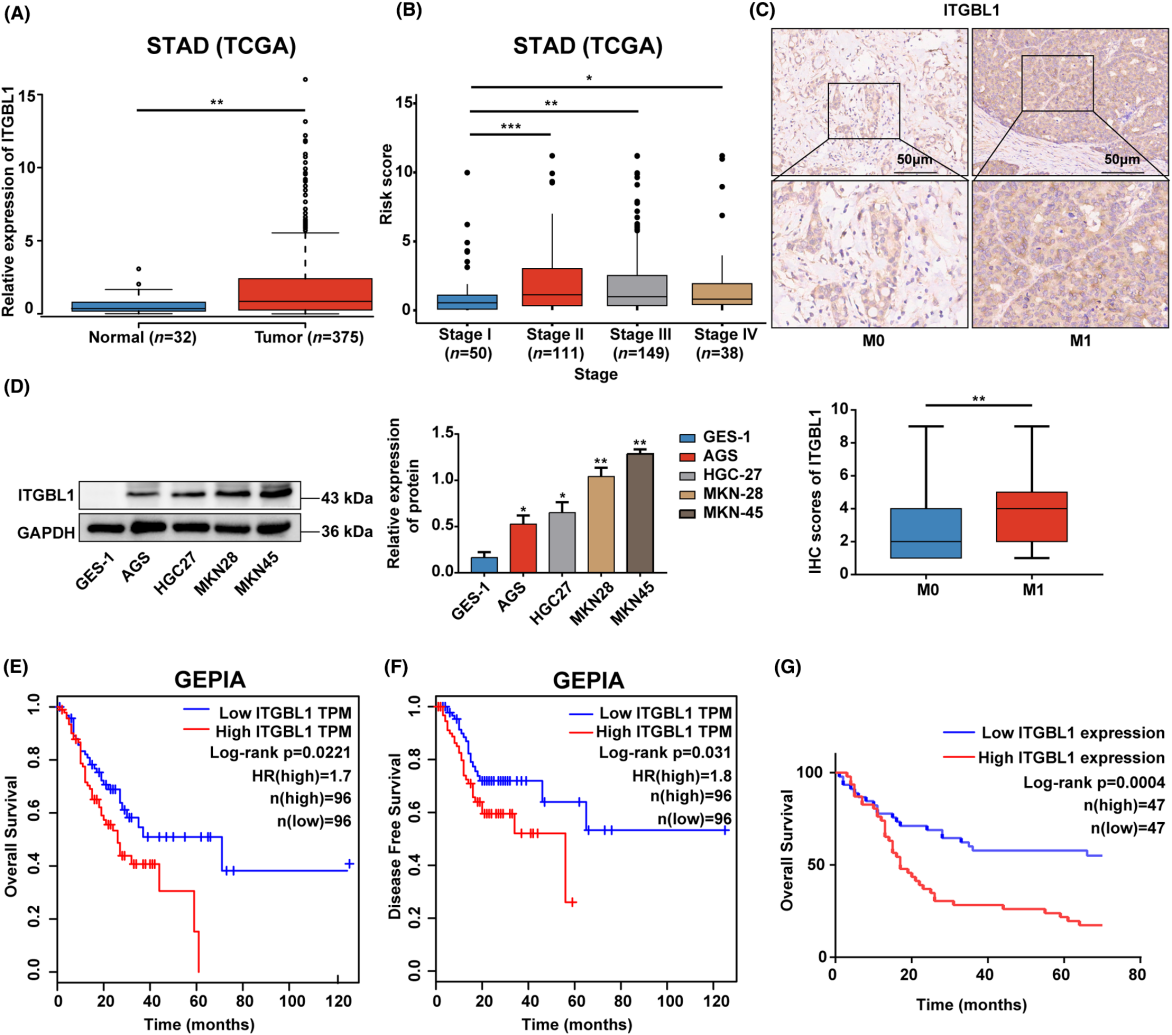
ITGBL1 expression was increased in GC tissues, and increased ITGBL1 expression was related to poor prognosis in GC patients (A). ITGBL1 expression in GC and normal gastric tissues based on the TCGA database. (B) Relationship between ITGBL1 expression and clinical stage in GC based on the TCGA database. (C) Typical images of stage M0 and M1 GC patients' tissues stained with ITGBL1 by IHC. Scale bar, 50 μm. (D) ITGBL1 protein expression in GES‐1, AGS, HGC27, MKN28 and MKN45 cells was measured by western blotting. GAPDH served as a loading control. The quantification of Western blot band densities was performed using the ImageJ program. (E–F) Kaplan–Meier curves of overall survival (log‐rank *p* = 0.0221) and disease‐free survival (log‐rank *p* = 0.031) of GC patients represented in the GEPIA database. (G) Kaplan–Meier curves of overall survival of GC patients represented in the TMAs (log‐rank *p* = 0.0004). The experiments were conducted in triplicate. The values were represented as means with standard deviations (SD), and the statistical significance was assessed using Student's *t*‐test. Nonsignificant results were denoted as ‘ns’, while significance levels were shown as *p < 0.05, **p < 0.01, and ***p < 0.001.

As shown in Figure 1A, through analysis of the TCGA database, we found that the mRNA levels of ITGBL1 in GC tissues exhibited a significant increase in comparison to the levels observed in normal gastric tissues (Figure 1A). Furthermore, the expression of ITGBL1 demonstrated a significant correlation with the TNM stage in GC (Figure 1B). Additionally, an IHC assay was utilized to evaluate ITGBL1 protein expression in clinical samples. The results showed that ITGBL1 expression was significantly higher among patients diagnosed with stage M1 disease compared to those diagnosed with stage M0 disease (Figure 1C). Furthermore, there was a strong correlation observed between the expression of ITGBL1 and the N stage in patients with GC (Table 1). We also measured ITGBL1 expression in cell lines, as shown in Figure 1D, and its expression in human gastric epithelial cell line GES‐1 was significantly lower than that in GC cells (AGS, HGC27, MKN28 and MKN45). We next analysed the relationship between ITGBL1 expression and prognosis in GC. In the GEPIA database, GC patients with high ITGBL1 mRNA expression experienced reduced overall survival and disease‐free survival durations. (Figure 1E,F). More importantly, Kaplan–Meier survival analysis revealed that high protein expression of ITGBL1 was significantly associated with poor prognosis in GC patients (Figure 1G).

**TABLE 1:** ITGBL1 expression and clinical features in grastric cancer patient samples.

Characteristic	ITGBL1 expression		*p*
	Low	High	
Gender			
Male	28	31	0.610
Female	18	16	
Age			
<60	16	10	0.147
>60	30	37	
T			
T1‐2	9	7	0.551
T3‐4	37	40	
N			
N0	16	6	0.012*
N1‐3	30	41	
M			
M0	46	46	1.0
M1	0	1	
AJCC stage			
I‐II	18	17	0.768
III‐IV	28	30	

*p < 0.05

### 3.2 Anoikis‐resistant GC cells were generated

Based on the expression levels of ITGBL1 in the four above mentioned GC cell lines, AGS cells, with relatively low levels of ITGBL1, and MKN45 cells, with relatively high levels of ITGBL1, were chosen to establish two anoikis‐resistant GC cell models. (Figure 1D). Compared with those in the corresponding wild‐type (WT) cells, the levels of cleaved PARP1 (CL‐PARP1), cleaved Caspase9 (CL‐Caspase9), cleaved Caspase3 (CL‐Caspase3) and BAX, four key pro‐apoptotic proteins, experienced a significant reduction in AR‐AGS and AR‐MKN45 cells under detachment culture conditions (Figure S1A). In contrast, the anti‐apoptotic protein Bcl‐2 levels were markedly increased in AR‐AGS and AR‐MKN45 cells (Figure S1A). The apoptosis assay results showed that the apoptosis rates of AR‐AGS and AR‐MKN45 cells were significantly lower than those of the corresponding WT cells after culture in suspension (Figure S1B). Furthermore, the results of Transwell experiments demonstrated notable enhancements in the migratory and invasive capacities of AR‐AGS and AR‐MKN45 cells (Figure S1C). Indeed, these results suggest that the AGS and MKN45 cell models of AR were successfully established.

### 3.3 ITGBL1 promoted the anoikis resistance and metastasis of GC cells

**FIGURE 2:**
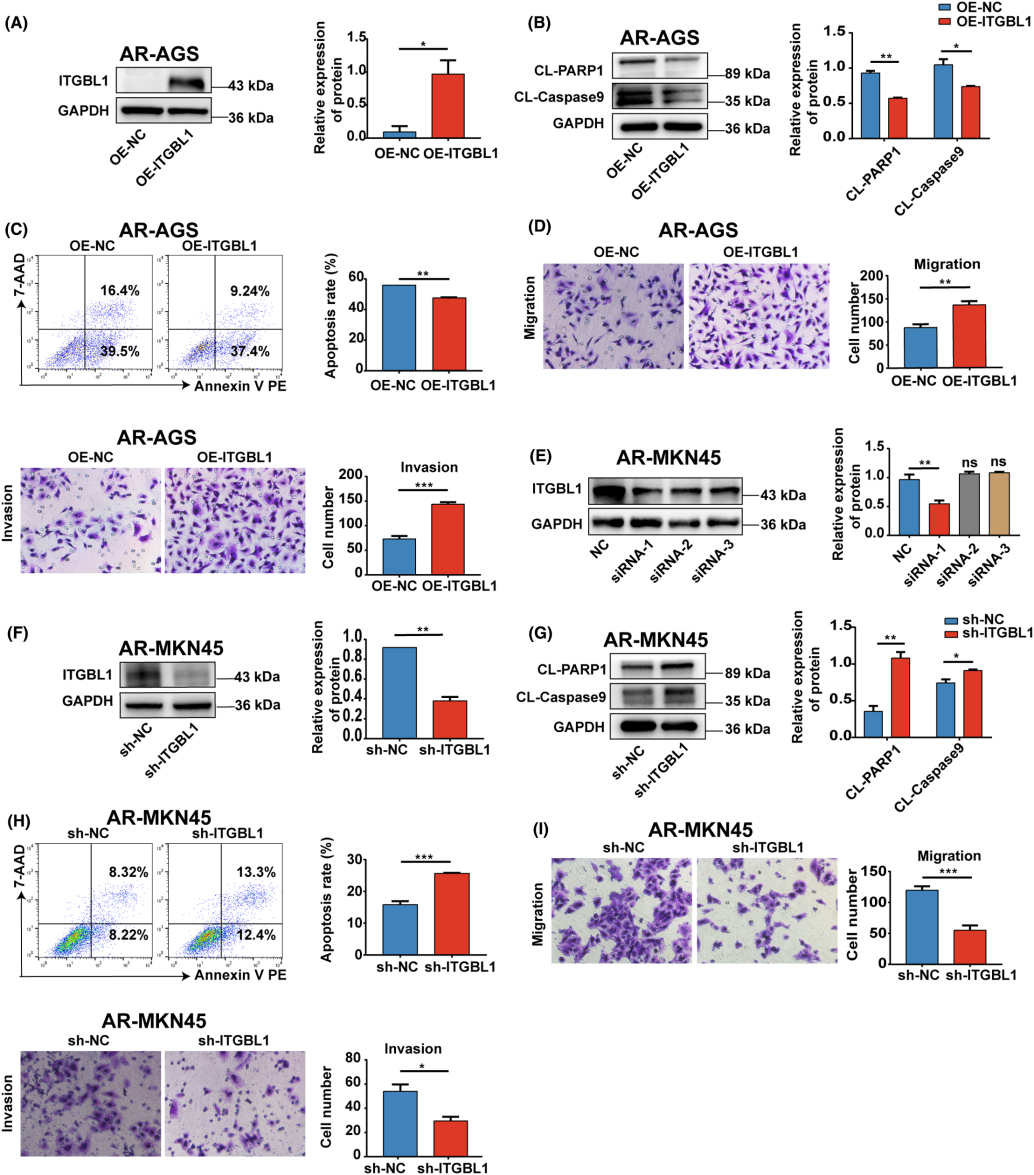
ITGBL1 promoted the anoikis resistance and metastasis of GC cells (A). Western blot analysis confirmed that ITGBL1 was overexpressed in AR‐AGS cells after lentiviral infection. GAPDH served as a loading control. The quantification of Western blot band densities was performed using the ImageJ program. (B) The protein levels of CL‐PARP1 and CL‐Caspase9 in AR‐AGS cells and ITGBL1‐overexpressing AR‐AGS cells after 48 h of suspension culture. (C) The apoptosis rate of AR‐AGS cells and ITGBL1‐overexpressing AR‐AGS cells after 48 h of suspension culture was determine by flow cytometry. (D) Transwell assays were used to measure the migration and invasion of AR‐AGS cells and ITGBL1‐overexpressing AR‐AGS cells. (E) ITGBL1 protein expression in AR‐MKN45 cells after transfection with ITGBL1 siRNAs. GAPDH served as a loading control. The quantification of Western blot band densities was performed using the ImageJ program. (F) The protein expression of ITGBL1 in AR‐MKN45 cells infected with lentivirus carrying the ITGBL1‐siRNA‐1 sequence. GAPDH served as a loading control. The quantification of Western blot band densities was performed using the ImageJ program. (G) The protein levels of CL‐PARP1 and CL‐Caspase9 in AR‐MKN45 cells and ITGBL1‐knockdown AR‐MKN45 cells after 48 h of suspension culture. (H) The apoptosis rate of AR‐MKN45 cells and ITGBL1‐knockdown AR‐MKN45 cells after 48 h of suspension culture was determined by flow cytometry. (I) Transwell assays were used to measure the migration and invasion of AR‐MKN45 cells and ITGBL1‐knockdown AR‐MKN45 cells. The experiments were conducted in triplicate. The values were represented as means with standard deviations (SD), and the statistical significance was assessed using Student's *t*‐test. Nonsignificant results were denoted as ‘ns’, while significance levels were shown as *p < 0.05, **p < 0.01, and ***p < 0.001.

To validate the roles of ITGBL1 in regulating anoikis resistance, stable ITGBL1‐overexpressing AR‐AGS cells were established using lentiviral transduction. The western blot results confirmed that the protein levels of ITGBL1 were significantly increased in ITGBL1‐overexpressing AR‐AGS cells compared with control cells (Figure 2A). Western blot analysis revealed marked decreases in the levels of CL‐PARP1 and CL‐Caspase9 in ITGBL1‐overexpressing AR‐AGS cells upon culture in suspension (Figure 2B). Compared with control cells, ITGBL1‐overexpressing AR‐AGS cells showed a significant decrease in the detachment‐induced apoptosis rate (Figure 2C). Furthermore, the migratory and invasive capacities of AR‐AGS cells overexpressing ITGBL1 were dramatically augmented (Figure 2D).

For complementary loss‐of‐function studies, three nonoverlapping siRNAs targeting ITGBL1 were employed to knock down ITGBL1 expression in AR‐MKN45 cells. Out of the three ITGBL1 siRNAs, ITGBL1 siRNA‐1 demonstrated the most pronounced inhibitory effect on the expression of ITGBL1 in AR‐MKN45 cells (Figure 2E), and this sequence was thus used to construct the ITGBL1 shRNA lentivirus (shITGBL1). AR‐MKN45 cells with stable ITGBL1 knockdown were established (Figure 2F). AR‐MKN45 cells with ITGBL1 knockdown showed significant increases in the detachment‐induced levels of CL‐PARP1 and CL‐Caspase9 and the apoptosis rate compared to those in control cells (Figure 2G,H). Furthermore, ITGBL1 knockdown significantly reduced the migration and invasion of AR‐MKN45 cells. (Figure 2I).

### 3.4 FBLN2 is negatively regulated by ITGBL1

**FIGURE 3:**
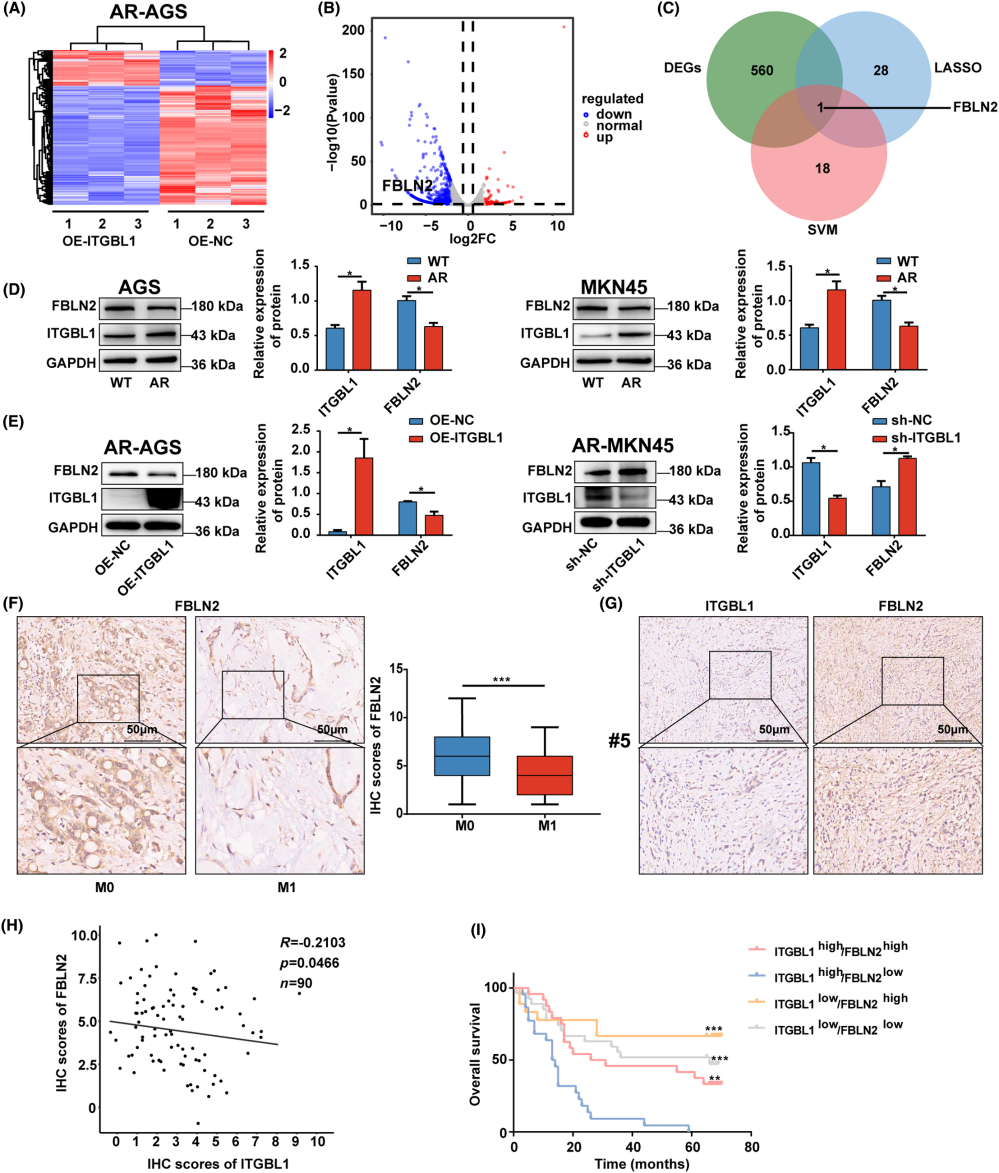
ITGBL1 negatively regulated FBLN2 expression (A). Heatmap of DEGs in AR‐AGS and ITGBL1‐overexpressing AR‐AGS cells after 48 h of suspension culture. (B) Volcano plot of DEGs in AR‐AGS and ITGBL1‐overexpressing AR‐AGS cells after 48 h of suspension culture. (C) Overlap analysis of DEGs between the LASSO technique and SVM algorithm. (D) The protein expression of FBLN2 and ITGBL1 in WT and AR‐GC cells. GAPDH served as a loading control. The quantification of Western blot band densities was performed using the ImageJ program. (E) The protein expression of FBLN2 in ITGBL1‐overexpressing AR‐AGS cells and ITGBL1‐knockdown AR‐MKN45 cells after 48 h of suspension culture. GAPDH served as a loading control. The quantification of Western blot band densities was performed using the ImageJ program. (F) Typical images of stage M0 and M1 GC patients' tissues stained with FBLN2 by IHC. Scale bar, 50 μm. (G) Typical images of ITGBL1 and FBLN2 IHC staining in tissue from GC patient #5 are shown. Scale bar, 50 μm. (H) Correlation analysis between ITGBL1 and FBLN2 expression in GC tissues (R = −0.2103, *p* = 0.0466). (I) Kaplan–Meier curves of overall survival of GC patients classified into four subgroups based on ITGBL1 and FBLN2 expression. The experiments were conducted in triplicate. The values were represented as means with standard deviations (SD), and the statistical significance was assessed using Student's *t*‐test. Nonsignificant results were denoted as ‘ns’, while significance levels were shown as *p < 0.05, **p < 0.01, and ***p < 0.001.

In order to investigate the potential mechanism underlying the phenomenon of ITGBL1‐mediated resistance to anoikis in AR‐GC cells, we first performed RNA‐seq analysis to profile the transcriptome in ITGBL1‐overexpressing AR‐AGS cells in suspension culture. Based on the RNA‐seq results, 561 differentially expressed genes, namely, 489 downregulated genes and 72 upregulated genes (*p* < 0.05 and |log2 (fold change)|>2), were identified in ITGBL1‐overexpressing AR‐AGS cells (Figure 3A,B). To better identify the downstream molecules regulated by ITGBL1, we conducted correlation analysis using the TCGA database to identify genes correlated with ITGBL1 in GC. As shown in Figure S2A, the patients with GC were categorized into two groups, namely a high expression group (*n* = 187) and a low expression group (*n* = 188), based on the median relative expression of ITGBL1 mRNA, with a cut‐off value of 0.92166570, and differential expression analysis was performed to identify differentially expressed genes, with 237 differentially expressed genes identified. Based on the WGCNA results, we concluded that the light‐yellow module containing 225 key genes, was the module most relevant to ITGBL1 expression (Figure S2B). By taking the intersection of the differentially expressed genes identified by differential expression analysis and those identified by WGCNA, a total of 59 overlapping ITGBL1‐related key genes were identified (Figure S2C). Then, we applied the LASSO technique (Figure S2D) and the SVM algorithm (Figure S2E) for analysis of these 59 differentially expressed genes and identified 26 and 19 genes, respectively, by these techniques. Notably, FBLN2 was present in all three differentially expressed gene datasets (Figure 3C), suggesting that FBLN2 was the most likely downstream gene regulated by ITGBL1. To verify this hypothesis, western blot analysis was performed, and the results showed that there was indeed a reverse relationship between ITGBL1 and FBLN2 expression in WT and AR‐GC cells (Figure 3D). Moreover, we observed that the protein level of FBLN2 in ITGBL1‐overexpressing AR‐AGS cells was significantly decreased, while the expression of FBLN2 in ITGBL1 knockdown AR‐MKN45 cells was significantly increased (Figure 3E). As shown in Figure 3F, FBLN2 expression was found to be significantly lower in patients with stage M1 disease than in those with stage M0 disease. Moreover, there was a negative correlation between FBLN2 and ITGBL1 expression in GC tissues (Figure 3G,H). Furthermore, the data in Table 2 demonstrate the close association between FBLN2 expression and T stage in GC patients (*p* = 0.047). Additionally, we divided the patients into four groups based on the ITGBL1 and FBLN2 expression levels: ITGBL1^high^FBLN2^high^, ITGBL1^high^FBLN2^low^, ITGBL1^low^FBLN2^high^ and ITGBL1^low^FBLN2^low^. The results from the Kaplan–Meier survival analysis revealed that patients in the ITGBL1^high^FBLN2^low^ group had a significantly worse prognosis than those in the other three groups (Figure 3I). Both univariate and multivariate regression models demonstrated that a high T stage, high N stage, high AJCC stage, high level of ITGBL1 expression and low level of FBLN2 expression were risk factors for overall survival in GC patients (Table 3). These findings strongly indicate that ITGBL1 might inversely regulate FBLN2 in GC.

**TABLE 2:** FBLN2 expression and clinical features in grastric cancer patient samples.

Characteristic	FBLN2 expression		*p*
	Low	High	
Gender			0.756
Male	31	28	
Female	19	15	
Age			0.963
<60	13	11	
>60	37	32	
T			0.047*
T1‐2	5	12	
T3‐4	45	31	
N			0.933
N0	12	10	
N1‐3	38	33	
M			1.0
M0	49	43	
M1	1	0	
AJCC stage			0.726
I‐II	18	17	
III‐IV	32	26	

*p < 0.05.

**TABLE 3:** Univariate and multivariate analysis of overall survival.

Characteristic	Univariate HR(95%CI)	*p*	Characteristic	Multivariate HR(95%CI)	*p*
Gender Male vs. Female	0.685(0.530–1.517)	0.685	Gender Male vs. Female	0.826(0.473–1.444)	0.503
Age ≥60 vs. <60	1.227(0.683–2.202)	0.494	Age ≥60 vs. <60	1.144(0.615–2.126)	0.671
T stage T3/T4 vs. T1/T2	3.328(1.139–8.338)	0.010*	T stage T3/T4 vs. T1/T2	2.220(0.867–5.680)	0.096
N stage N1/N2/N3 vs. N0	4.776(2.046–11.147)	<0.001***	N stage N1/N2/N3 vs. N0	3.537(1.465–8.542)	0.005**
AJCC stage III/IV vs. I/II	3.317(1.833–6.004)	<0.001***	ITGBL1 expression high vs. low	2.390(1.311–4.356)	0.004**
ITGBL1 expression high vs. low	2.498(1.459–4.297)	0.001**	FBLN2 expression high vs. low	0.502(0.290–0.869)‐	0.014*
FBLN2 expression high vs. low	0.573(0.340–0.96)	0.037*			

*p < 0.05, **p < 0.01, ***p < 0.001.

### 3.5 FBLN2 was involved in ITGBL1‐mediated anoikis resistance and metastasis in GC cells

**FIGURE 4:**
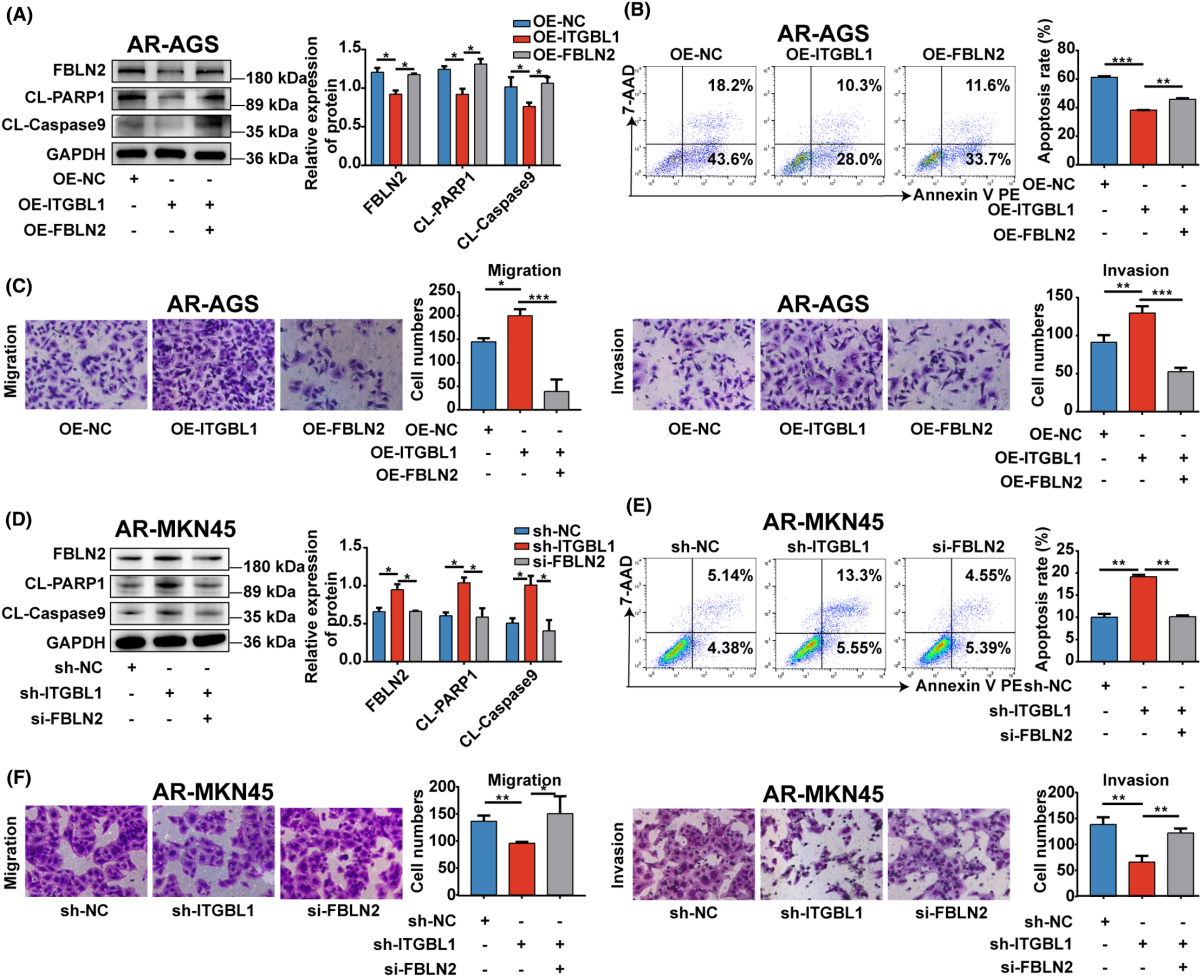
FBLN2 was involved in ITGBL1‐mediated anoikis resistance and metastasis in GC cells (A). The protein levels of FBLN2, CL‐PARP1 and CL‐Caspase9 in ITGBL1‐overexpressing AR‐AGS cells after treatment with the FBLN2 overexpression plasmid. GAPDH served as a loading control. The quantification of Western blot band densities was performed using the ImageJ program. (B) The apoptosis rate of ITGBL1‐overexpressing AR‐AGS cells after treatment with the FBLN2 overexpression plasmid was determined by flow cytometry. (C) Transwell assays were used to measure the migration and invasion of ITGBL1‐overexpressing AR‐AGS cells treated with the FBLN2 overexpression plasmid. (D) The protein levels of FBLN2, CL‐PARP1 and CL‐Caspase9 in ITGBL1‐knockdown AR‐MKN45 cells treated with FBLN2 siRNA. (E) The apoptosis rate of ITGBL1‐knockdown AR‐MKN45 cells after treatment with FBLN2 siRNA was determined by flow cytometry. (F) Transwell assays were used to measure the migration and invasion of ITGBL1‐knockdown AR‐MKN45 cells after treatment with FBLN2 siRNA. The experiments were conducted in triplicate. The values were represented as means with standard deviations (SD), and the statistical significance was assessed using Student's *t*‐test. Nonsignificant results were denoted as ‘ns’, while significance levels were shown as *p < 0.05, **p < 0.01, and ***p < 0.001.

To assess whether ITGBL1‐mediated anoikis resistance and metastasis are dependent on FBLN2, a plasmid carrying FBLN2 cDNA was introduced into ITGBL1‐overexpressing AR‐AGS cells. As shown in Figure 4A,B, FBLN2 overexpression significantly increased the protein levels of FBLN2 in ITGBL1‐overexpressing AR‐AGS cells, which were initially decreased in ITGBL1‐overexpressing AR‐AGS cells. Overexpression of FBLN2 reversed the effect of ITGBL1 overexpression on the detachment‐induced decreases in the protein levels of CL‐PARP1 and CL‐Caspase9 and the apoptosis rate in AR‐AGS cells. The results of Transwell assays also demonstrated that the increases in the migration and invasion abilities induced by ITGBL1 overexpression were suppressed by FBLN2 overexpression in AR‐AGS cells (Figure 4C). Rescue experiments were also performed in ITGBL1 knockdown AR‐MKN45 cells by transfecting a commercial FBLN2 siRNA, which reduced FBLN2 expression in ITGBL1 knockdown AR‐MKN45 cells (Figure 4D). Consistent with our prediction, knockdown of ITGBL1 resulted in increases in the protein levels of CL‐PARP1 and CL‐Caspase9 and the apoptosis rate and decreases in the migration and invasion of AR‐MKN45 cells, and all of these effects were reversed by silencing of FBLN2 (Figure 4D–F). In summary, these results reveal that ITGBL1 enhances the anoikis resistance and metastasis of GC cells via FBLN2.

### 3.6 ITGBL1 modulated FBLN2 expression in GC cells and promoted their anoikis resistance and metastasis through the AKT signalling pathway

**FIGURE 5:**
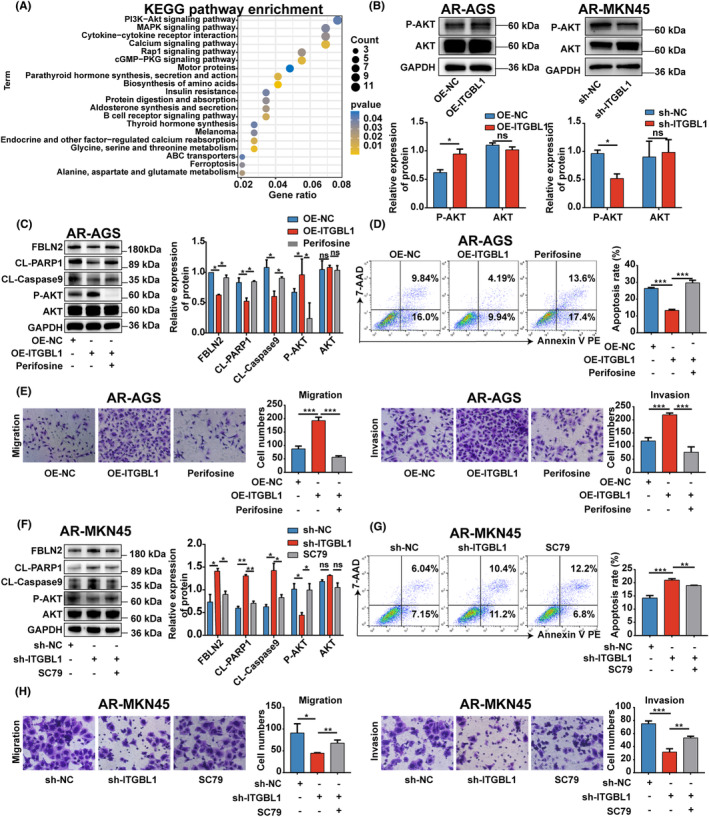
ITGBL1 modulated FBLN2 expression in GC cells and their anoikis resistance and metastasis through the AKT signalling pathway (A). KEGG pathway enrichment analysis of DEGs in AR‐AGS and ITGBL1‐overexpressing AR‐AGS cells after 48 h of suspension culture. (B) The protein levels of P‐AKT and AKT in ITGBL1‐overexpressing AR‐AGS cells and ITGBL1‐knockdown AR‐MKN45 cells after 48 h of suspension culture. GAPDH served as a loading control. The quantification of Western blot band densities was performed using the ImageJ program. (C) The protein levels of FBLN2, CL‐PARP1, CL‐Caspase9, P‐AKT, and AKT in ITGBL1‐overexpressing AR‐AGS cells treated with perifosine. GAPDH served as a loading control. The quantification of Western blot band densities was performed using the ImageJ program. (D) The apoptosis rate of ITGBL1‐overexpressing AR‐AGS cells after treatment with perifosine was determined by flow cytometry. (E) Transwell assays were used to measure the migration and invasion of ITGBL1‐overexpressing AR‐AGS cells treated with perifosine. (F) The protein levels of FBLN2, CL‐PARP1, CL‐Caspase9, P‐AKT and AKT in ITGBL1‐knockdown AR‐MKN45 cells treated with SC79. GAPDH served as a loading control. The quantification of Western blot band densities was performed using the ImageJ program. (G) The apoptosis rate of ITGBL1‐knockdown AR‐MKN45 cells after treatment with SC79 was determined by flow cytometry. (H) Transwell assays were used to measure the migration and invasion of ITGBL1‐knockdown AR‐MKN45 cells after treatment with SC79. The experiments were conducted in triplicate. The values were represented as means with standard deviations (SD), and the statistical significance was assessed using Student's *t*‐test. Nonsignificant results were denoted as ‘ns’, while significance levels were shown as *p < 0.05, **p < 0.01, and ***p < 0.001.

To investigate how ITGBL1 regulates FBLN2 expression in AR‐GC cells, we performed KEGG enrichment analysis on the previously obtained differentially expressed genes in ITGBL1‐overexpressing AR‐AGS cells. The results of KEGG enrichment analysis showed that upregulation of ITGBL1 significantly affected the AKT signalling pathway, and FBLN2 is also in the AKT pathway (Figure 5A). Therefore, we hypothesized that ITGBL1 might inhibit the expression of FBLN2 through the AKT pathway in AR‐GC cells. The Western blot results showed that the protein level of P‐AKT in ITGBL1‐overexpressing AR‐AGS cells was significantly increased, while the expression of P‐AKT in ITGBL1 knockdown AR‐MKN45 cells was significantly decreased (Figure 5B). Then, perifosine, an inhibitor of the AKT pathway, was used to demonstrate whether ITGBL1 inhibits FBLN2 expression in GC cells and promotes anoikis resistance and metastasis through the AKT signalling pathway. As shown in Figure 5C,D, treatment with perifosine abolished the inhibitory effect of ITGBL1 overexpression on FBLN2 expression in AR‐AGS cells. Furthermore, we observed that the application of perifosine resulted in the reversal of the impacts caused by ITGBL1 overexpression on the protein levels of CL‐PARP1 and CL‐Caspase9, as well as the rate of apoptosis in AR‐AGS cells. In addition, the overexpression of ITGBL1 significantly enhanced the migratory and invasive capabilities of AR‐AGS cells. However, this effect was effectively counteracted by the administration of perifosine (Figure 5E). Importantly, these results were further validated in ITGBL1 knockdown AR‐MKN45 cells by using SC79, an activator of the AKT pathway (Figure 5F–H). Thus, these data suggest that ITGBL1 suppresses FBLN2 expression in AR‐GC cells and enhances anoikis resistance and metastasis through the AKT signalling pathway.

### 3.7 The ITGBL1/FBLN2 axis promoted lung metastasis of AR‐GC cells in vivo

**FIGURE 6:**
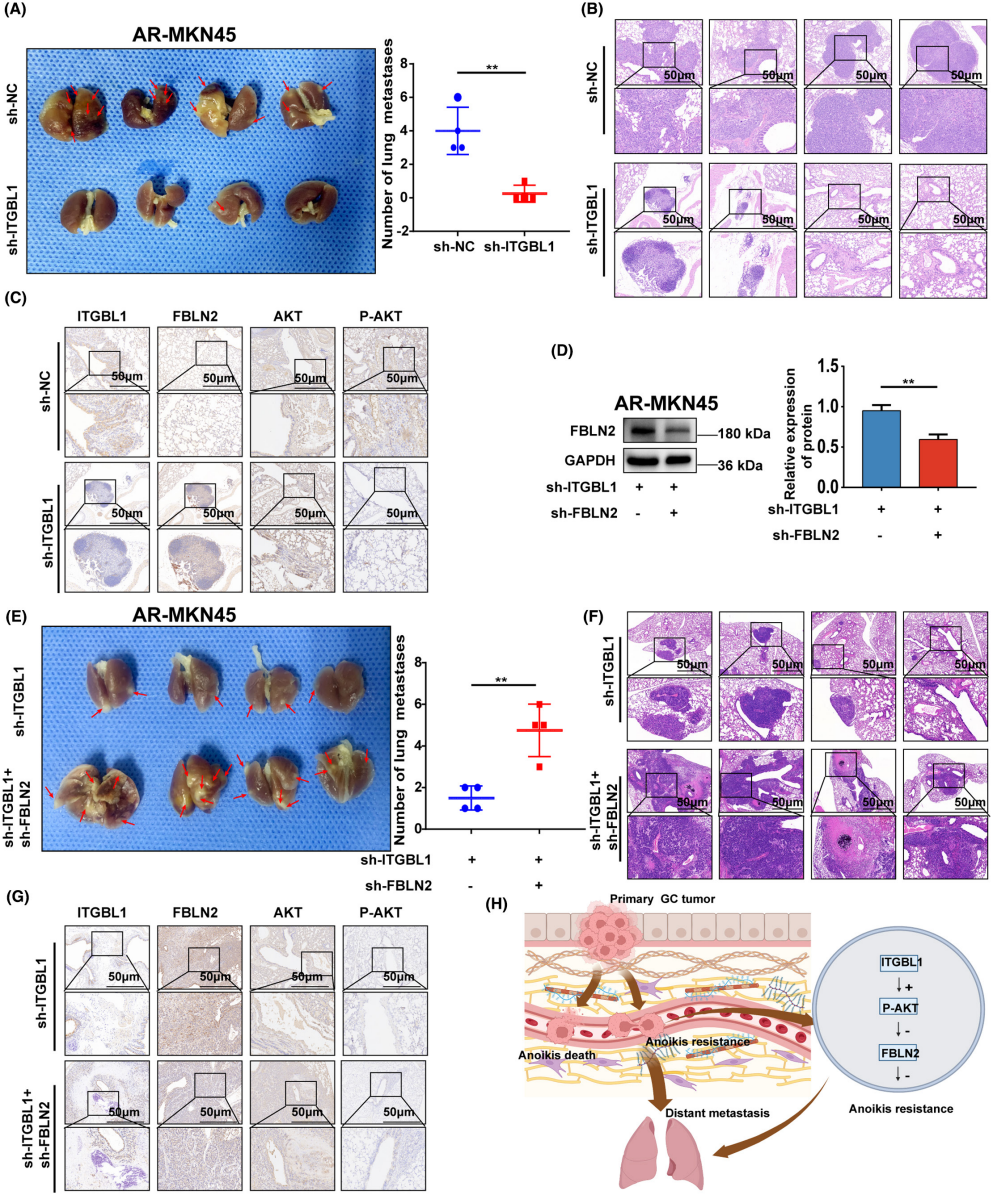
The ITGBL1/FBLN2 axis promoted lung metastasis of AR‐GC cells in vivo. (A) Images of mouse lungs after transplantation with AR‐MKN45 cells and ITGBL1‐knockdown AR‐MKN45 cells. (B) Typical images of haematoxylin–eosin staining in lung metastases from the sh‐NC and sh‐ITGBL1 groups. Scale bar, 50 μm. (C) The expression of ITGBL1, FBLN2, AKT, and P‐AKT in mouse lung tissues in the sh‐NC group and the sh‐ITGBL1 group was determined by IHC experiments. Scale bar, 50 μm. (D) The protein expression of FBLN2 in ITGBL1‐knockdown AR‐MKN45 cells after infection with lentivirus carrying FBLN2 siRNA. GAPDH served as a loading control. The quantification of Western blot band densities was performed using the ImageJ program. (E) Images of mouse lungs after transplantation with ITGBL1‐knockdown AR‐MKN45 cells and ITGBL1/FBLN2 double‐knockdown AR‐MKN45 cells. (F) Typical images of haematoxylin–eosin staining in lung metastases from the sh‐ITGBL1 and sh‐ITGBL1 + sh‐FBLN2 groups. Scale bar, 50 μm. (G) The expression of ITGBL1, FBLN2, AKT, and P‐AKT in mouse lung tissues in the sh‐ITGBL1 group and the sh‐ITGBL1 + sh‐FBLN2 group was determined by IHC experiments. Scale bar, 50 μm. (H) Diagrammatic representation of the ITGBL1/AKT/FBLN2 axis's regulation mechanisms in GC anoikis resistance and metastasis. The experiments were conducted in triplicate. The values were represented as means with standard deviations (SD), and the statistical significance was assessed using Student's *t*‐test. Nonsignificant results were denoted as ‘ns’, while significance levels were shown as *p < 0.05, **p < 0.01, and ***p < 0.001.

To evaluate the effect of the ITGBL1/FBLN2 axis on the metastasis of AR‐GC cells in vivo, a mouse lung metastasis model was established using AR‐MKN45 cells injected via the tail vein. As shown in Figure 6A,B, compared with that in the sh‐NC group, the number of metastatic nodules in the lung tissues of mice in the sh‐ITGBL1 group was significantly reduced. Then, the expression of ITGBL1, FBLN2, AKT, and P‐AKT in mouse lung tissues was examined by IHC. The results showed that the expression of ITGBL1 and P‐AKT was significantly decreased in the sh‐ITGBL1 group; while the expression of FBLN2 was markedly increased in the sh‐ITGBL1 group (Figure 6C). To demonstrate whether ITGBL1 modulates lung metastasis of AR‐GC cells in vivo by downregulating FBLN2 expression, ITGBL1/FBLN2 double‐knockdown AR‐MKN45 cells were generated and used to establish a mouse lung metastasis model (Figure 6D). Compared with that in the ITGBL1 knockdown group, there was a considerable increase observed in the quantity of lung metastatic nodules in the ITGBL1/FBLN2 double‐knockdown group (Figure 6E,F). Furthermore, FBLN2 knockdown decreased the expression of FBLN2 in the ITGBL1/FBLN2 double‐knockdown group, but had no influence on the expression of ITGBL1 and P‐AKT (Figure 6G). These results suggest that ITGBL1 promotes lung metastasis of AR‐GC cells in vivo by negatively regulating FBLN2.

## 4 DISCUSSION

Many investigations have been conducted to examine the biological roles of ITGBL1 in numerous primary tumours and cancer cell lines.^14^, ^18^, ^19^ For example, ITGBL1 can inhibit the tumoricidal ability of NK cells and promote the occurrence and development of melanoma.^20^ Another investigation revealed that the primary tumours secrete extracellular vesicles abundant in ITGBL1, which facilitate alterations in the milieu of fibrocytes located in remote organs, ultimately fostering their metastatic progression.^19^ In HCC, the upregulation of ITGBL1 induces enhanced migratory and invasive capabilities in HCC cells through the activation of the TGF‐β/Smad pathway.^11^ Nevertheless, there is a paucity of research examining the functions of ITGBL1 in GC. According to a bioinformatics analysis‐based study, it was shown that the overexpression of ITGBL1 had an impact on the overall survival.^21^ A study by Yin et al. shown that the levels of RNA and protein of ITGBL1 were elevated in samples from patients with gastric cancer when compared to the neighbouring non‐tumour tissues.^15^ In this study, an elevation in the expression of ITGBL1 was noted in GC patients diagnosed with stage M1 disease in comparison to those diagnosed with stage M0 disease. Moreover, upregulation of ITGBL1 was associated with a poor prognosis. These clinical findings presented in this study indicate that ITGBL1 may possess a significant role in the process of GC metastasis. Moreover, our study provides evidence that ITGBL1 has a greater propensity to influence the resistance of AR‐GC cells to anoikis, thereby leading to alterations in their metastatic potential.

Anoikis resistance exhibits a strong correlation with the processes of metastasis and tumour growth.^22^, ^23^ Thus, the identification of crucial regulators responsible for conferring resistance to anoikis, as well as understanding the underlying mechanisms via which their dysregulation occurs, holds significant importance in cancer progression. Jin et al. showed that GDH1, a glutaminolytic enzyme, which involved in energy generation resulted in the acquisition of anoikis resistance.^24^ In prostate cancer, circCEMI enhanced anoikis resistance by promoting transmembrane 9 superfamily member 4 (TM9SF4)‐mediated protective autophagy.^25^ Our current study represents the first demonstration of the pivotal role played by ITGBL1 in governing the processes of anoikis and metastasis in AR‐GC cells. This provides novel evidence of the mechanism by which GC cells adapt to the external environment for anoikis avoidance and promotion of metastasis.

Fibulin‐2 (FBLN2) is an extracellular glycoprotein and was initially discovered in embryonic endocardial cushion tissue and adult mouse and human heart valves, and FBLN2 may be involved in tissue development and remodelling.^26^, ^27^ According to reports, FBLN2 has been identified to exhibit tumour suppressor properties in several cancer types, such as breast cancer,^28^ non‐small cell lung cancer,^29^ and nasopharyngeal carcinoma.^30^ In contrast, FBLN2 was reported to act as an oncogene in urothelial carcinoma^31^ and colorectal cancer.^32^ Therefore, the above studies suggest that the biological functions of FBLN2 are associated with cancer type. In the current study, we observed a negative correlation between ITGBL1 protein expression and FBLN2 protein expression in AR‐GC cell lines and GC cancer tissues. Moreover, the upregulation of FBLN2 counteracted the impact of ITGBL1 upregulation on the resistance of AR‐GC cells to anoikis, as well as their migratory and invasion capabilities. Furthermore, FBLN2 knockdown also demonstrated this negative correlation. The aforementioned findings indicate that ITGBL1 plays a significant role in enhancing the resistance of AR‐GC cells to anoikis and facilitating their metastatic capabilities, with these effects being dependent on the presence of FBLN2.

The active AKT signalling pathway is widely recognized as a classical prosurvival system that has significant influence over various cellular activities, including but not limited to normal cell survival, growth, proliferation, angiogenesis, transcription, translation, and metabolism.^33^, ^34^ Furthermore, the involvement of the active AKT signalling pathway in malignancies has been revealed in relation to its role in anoikis resistance and metastasis.^35^ In a study conducted by Lin Li and colleagues, it was discovered that miR‐137 facilitated the process of pancreatic cancer cell anoikis. This effect was achieved by the targeting of paxillin, a protein that plays a role in the activation of the AKT signalling pathway.^36^ In the current study, KEGG enrichment analysis based on the previously identified differentially expressed genes in ITGBL1‐overexpressing AR‐AGS cells showed that the considerable impact of ITGBL1 overexpression on the AKT signalling pathway, and FBLN2 is in the AKT pathway. A study by Yin et al. indicated that the activation of the AKT signalling pathway, caused by ITGBL1, resulted in an increase in cell proliferation and invasion in GC.^15^ Hence, we hypothesized that ITGBL1 suppressed FBLN2 expression and promoted anoikis resistance and metastasis in GC via the AKT signalling pathway. The results of our rescue experiments demonstrated that the inhibitor perifosine and activator SC79 effectively counteracted the impacts of ITGBL1 overexpression and knockdown, respectively, on FBLN2 expression, resistance to anoikis, and metastasis in GC. These findings suggest that ITGBL1 can activate the AKT/FBLN2 axis, further enhancing anoikis resistance and metastasis in GC. However, the precise mechanism of ITGBL1‐induced AKT pathway activation remains unclear. Further investigation is needed in future studies.

In conclusion, we have successfully illustrated the distinct role of ITGBL1 in conferring resistance to anoikis and promoting metastasis in GC. Moreover, ITGBL1 upregulation led to an increase in anoikis resistance and metastasis in GC through the AKT/FBLN2 axis (Figure 6H). Therefore, therapeutic strategies based on inhibition of ITGBL1 to disrupt the anoikis resistance and metastasis of GC cells may be a promising approach for GC.

## 5 CONCLUSION

Our study presents an investigation into the novel role of ITGBL1 in anoikis resistance and metastasis of GC. The high expression of ITGBL1 in GC can promote tumour metastasis by promoting anoikis resistance, and ultimately affect the survival and prognosis of patients. This study reveals a previously unknown mechanism by which ITGBL1 can suppress FBLN2 expression through AKT signalling pathway. This study helps to improve the understanding of anoikis and metastasis of GC, and the ITGBL1/AKT/FBLN2 signalling pathway can be used as a potential therapeutic target.

## AUTHOR CONTRIBUTIONS

**Tongguo Shi:** Data curation (equal); formal analysis (equal); software (equal). **Kanger Shen:** Data curation (lead); funding acquisition (lead); visualization (equal); writing – review and editing (equal). **Wei Xia:** Funding acquisition (equal); supervision (equal). **Kun Wang:** Investigation (equal); validation (equal). **Juntao Li:** Investigation (equal); supervision (equal). **Wei Xu:** Visualization (equal); writing – original draft (equal). **Haoran Liu:** Formal analysis (equal); funding acquisition (equal). **Kexi Yang:** Validation (equal). **Jinghan Zhu:** Investigation (equal). **Jiayu Wang:** Formal analysis (equal). **Qinhua Xi:** Investigation (equal). **Rui Li:** Funding acquisition (equal).

## CONFLICT OF INTEREST STATEMENT

The authors have declared that no competing interest exists.

## Supporting information


Figure S1.

Figure S2.

Table S1:

Table S2:

Table S3.

## Data Availability

The data that support the findings of this study are available from the corresponding author upon reasonable request.
