# Supplementary figures and images for: ITGBL1 promotes anoikis resistance and metastasis in human gastric cancer via the AKT/FBLN2 axis
Source: J Cell Mol Med. 2024 Feb 8;28(4):e18113. doi: 10.1111/jcmm.18113 (PMC10853594; doi:10.1111/jcmm.18113)

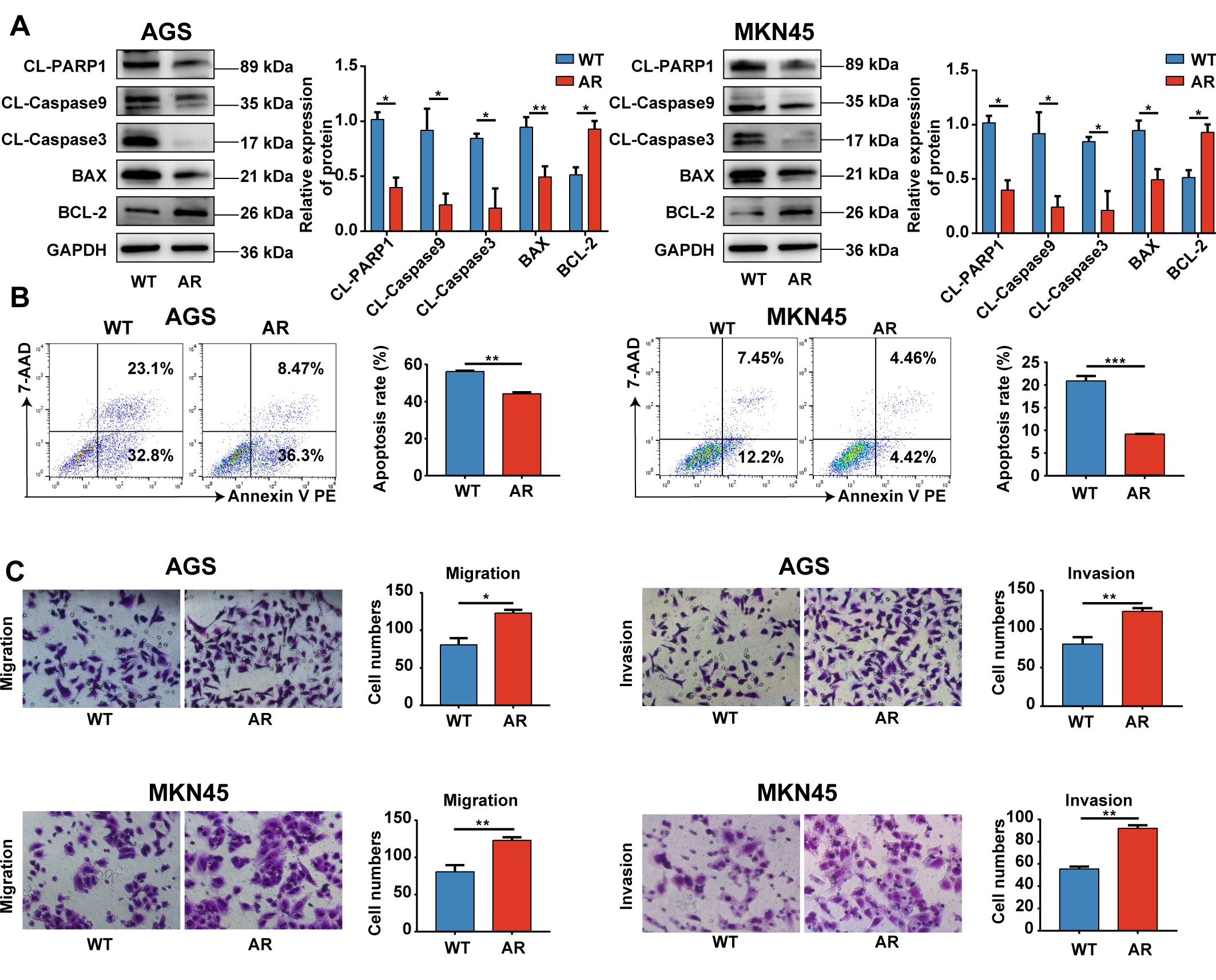

Supplement: Supplementary file 1 — Figure S1. [file JCMM-28-e18113-s001.tif]

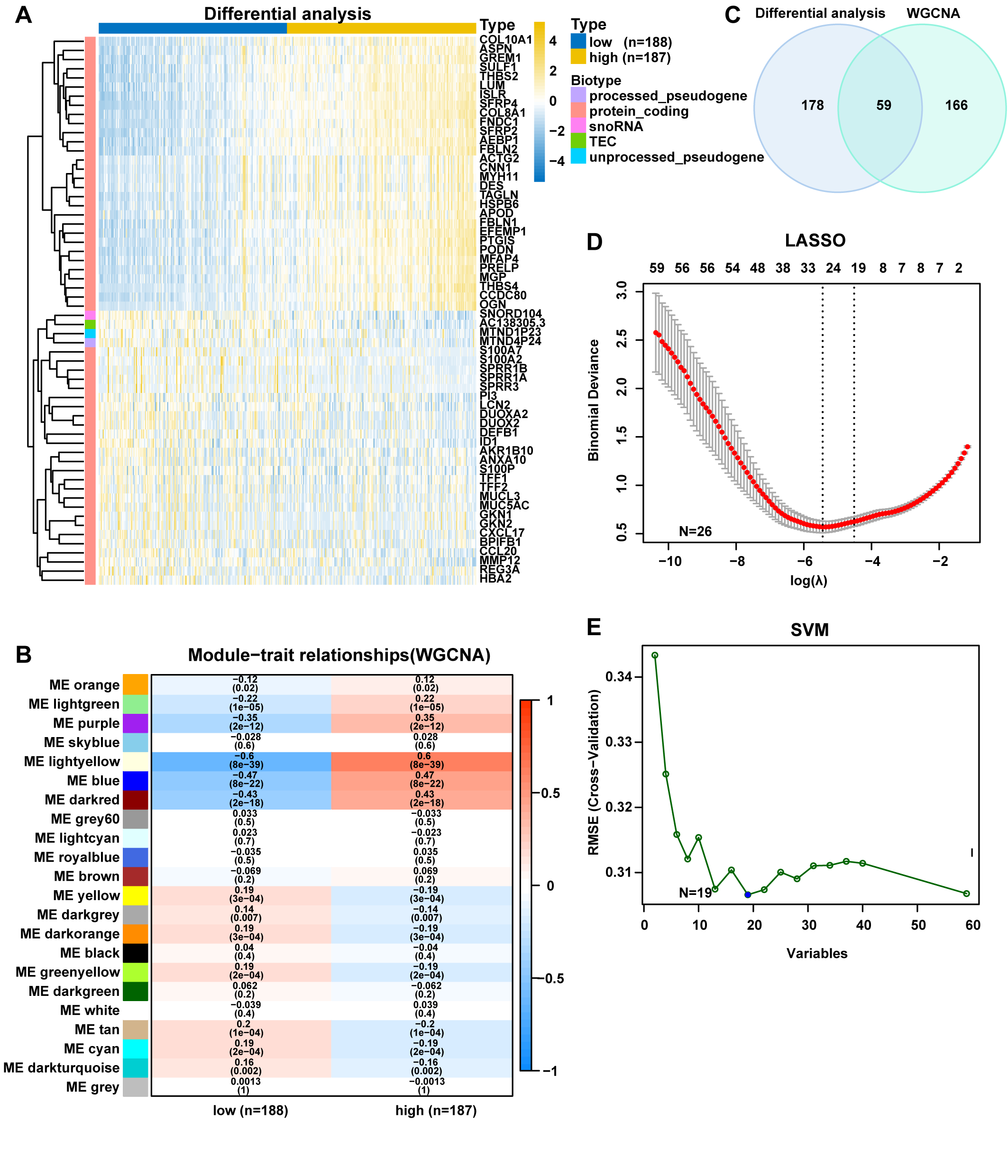

Supplement: Supplementary file 2 — Figure S2. [file JCMM-28-e18113-s005.tif]
